# Non-invasive diagnosis and monitoring tool of children’s mental health: A point-of-care immunosensor for IL-6 quantification in saliva samples

**DOI:** 10.3389/fnins.2022.919551

**Published:** 2022-09-26

**Authors:** Andrea Cruz, Maria Vieira, Ana R. Mesquita, Adriana Sampaio, Inês Mendes-Pinto, Isabel Soares, Paulo P. Freitas

**Affiliations:** ^1^ProChild CoLAB Against Child Poverty and Social Exclusion, Portuguese Foundation for Science and Technology (FCT) Collaborative Laboratory, Guimarães, Portugal; ^2^International Iberian Nanotechnology Laboratory, Braga, Portugal; ^3^CIPsi, School of Psychology, University of Minho, Braga, Portugal

**Keywords:** immunosensor, IL-6, saliva, children, mental health, point-of-care

## Abstract

Mental disorders are commonly featured as chronic conditions with often onset during childhood. In this context, inflammation has been associated with a higher risk of developing physical and mental health problems. Interleukin (IL)-6 is a key mediator of inflammatory responses and plays a pivotal role in immune and nervous system interaction. High levels of IL-6 during childhood are associated with mental problems, indicating that the IL-6 molecular pathway may represent a new target for monitoring and treating these conditions. Here, we report the detection of IL-6 in saliva samples from children (*N* = 118, mean age 4.4 years old) with behavioral problems using an immunosensor based on electrochemical impedance spectroscopy. This work demonstrates that the proposed immunosensor requires smaller sample volumes and is significantly faster and more sensitive than conventional ELISA while maintaining comparable levels of specificity and reproducibility. The point-of care immunosensor for detection of IL-6 in saliva samples presented herewith is, therefore, an attractive solution to the clinical practice as a rapid non-invasive, high-sensitive monitoring tool of mental health problems, especially in vulnerable patient populations such as children.

## Introduction

The human stress response is a natural defense mechanism to preserve and achieve a condition of homeostasis upon an adverse event (Gunnar and Quevedo, 2007). During a normal stress stimulation the hypothalamic-pituitary-adrenal (HPA) axis initiates the release of glucocorticoids into the bloodstream that regulate the levels of pro-and anti-inflammatory molecules. Through a negative feedback mechanism, these molecules control the release of these hormones via the HPA pathway, preventing an exacerbated inflammatory response (Hänsel et al., 2010; del Rey and Besedovsky, 2017). However, uncontrolled or chronic stress responses can disrupt this mechanism, resulting in harmful health effects. In the face of chronic stress or life adversity, there is a deregulation of this bidirectional communication, thus inducing a positive feedback loop, which increases inflammation (Brenhouse and Schwarz, 2016; Jiang et al., 2018). Therefore, affecting the functioning of neural circuits and negatively influences behavior (Miller et al., 2012; Mesquita et al., 2015; Calcia et al., 2016; Simons et al., 2019), and predisposes individuals to the development of psychopathologies (i.e., psychotic behavioral, depressive and anxiety disorders).

Recent studies have demonstrated that adversity during the first few years of life promotes inflammation throughout life, increasing the risk of developing physical and mental health problems (Fagundes and Way, 2014; Simons et al., 2019; Cuartas, 2020; Jiao et al., 2020; Loades et al., 2020). Several studies have provided evidence that childhood and adolescent exposure to adversity significantly correlates with elevated levels of inflammatory biomarkers such as C-reactive 3 protein (CRP) and interleukin (IL)-6 (Danese et al., 2009; Carpenter et al., 2010; Lacey et al., 2014; Raposa et al., 2015; D’Elia et al., 2018). The IL-6 is a pleiotropic cytokine that plays a vital role in interacting between the immune and nervous systems (Erta et al., 2012). While IL-6 has neurotrophic properties and beneficial effects in the central nervous system, its overexpression is associated with neurological, psychiatric and emotional disorders (Erta et al., 2012; Wei et al., 2012; Slopen et al., 2013; de Baumont et al., 2019). In fact, a growing body of evidence proposes that IL-6 has a central role in the pathogenesis of depression (Ting et al., 2020). Additionally, it has been demonstrated that IL-6 levels are associated with presence of internalization and externalization behaviors in children’s (Slopen et al., 2013; Tang et al., 2019), and that increase levels of the IL-6 in childhood are associated with an augmented risk of developing psychosis and depression in young adulthood (Khandaker et al., 2014; de Baumont et al., 2019; Perry et al., 2021). Therefore, scientific evidence suggests that IL-6 pathway may be a new target for diagnosing and treating mental disorders (de Baumont et al., 2019; Perry et al., 2021).

Typically, cytokines are measured using an enzyme-linked immunosorbent assay (ELISA). Conventional ELISA assays lack the ease of use and need trained personnel to perform, require high sample volumes that may be difficult to collect, are time consuming and relatively expensive, contributing to the difficulty of diagnosing diseases rapidly and making them unpractical for the purpose of monitoring cytokine-related diseases and/or conditions. Additionally, in normal conditions, cytokines are usually present at low pg/mL levels in body fluids, falling below the detection limits of ELISA assay, thereby affecting the quantitative analysis and reliable interpretation of results. Thus, to increase the detection accuracy of these biomarkers, there is a necessity to improve highly sensitive and easy-to-use technologies.

In line with this, saliva analysis provides numerous advantages over blood as a non-invasive alternative body fluid, including the ease of sample collection in vulnerable patient populations (i.e., children), lack of need for immediate sample processing, specialized instruments or trained personnel, ability to collect samples in different settings and minimal exposure of healthcare personnel to blood-borne pathogens (Rathnayake et al., 2013; Yoshizawa et al., 2013). In fact, an increasing number of biomarkers associated with local and systemic diseases have been detected in saliva for different pathologies, from oncological (Rapado-González et al., 2020), cardiovascular (Gohel et al., 2018), infectious (Estévez et al., 2011; Napodano et al., 2021) or neurological (Jasim et al., 2018; Corey-Bloom et al., 2020) diseases. Inflammatory molecules (i.e., IL-6, IL-8, tumor necrosis factor (TNF)-α) are among these identified biomarkers present in saliva (Prasad et al., 2016).

Overall, this highlights the relevance of quantifying IL-6 in saliva samples for neurological disease diagnosis and monitoring in children’s. Therefore, this work presents the validation of a label-free immunosensor based on the electrochemical impedance spectroscopy (EIS) system for a non-invasive quantitative detection of IL-6 in saliva samples.

## Materials and methods

### Immunosensor design and functionalization

Biomarker quantification was performed using gold screen-printed electrodes (SPEs) (DropSens, C223AT) with a printed gold working electrode (WE) (1.6 mm ∅), a gold counter electrode (CE), and a silver pseudo reference electrode (RE) (Cruz et al., 2019, 2021). The gold-SPEs were pre-cleaned with isopropanol and deionized (DI) water, prior to functionalization. To form the self-assembled monolayer (SAM) the sensor was incubated for 20 min at room temperature (RT), with a solution (10 mg/mL) of sulfo-LC-SPDP (sulfosuccinimidyl 6-(3′-(2-pyridyldithio)propionamido)hexanoate) (Thermo Fisher) in 10 mM phosphate buffer (PB, pH7.4) with 5% glycerol and washed with PB. Anti-IL-6 antibodies (eBioscience#88-7066) (0.25 μg/μL antibody solution (in PB) with 5% glycerol), were linked to SAM through overnight incubation, at 4 °C, followed by rinsed with PB. Next sensors were incubated with 0.25% BSA solution in PB at RT for 30 min with 5% glycerol to block non-specific interactions and unreacted sites.

### Electrochemical impedance spectroscopy measurements

To characterize the gold WE surface in terms of electron transfer kinetics and redox processes, the Electrochemical Impedance Spectroscopy (EIS) analysis was performed during the electrode functionalization (Bariya et al., 2018). EIS used for IL-6 detection and quantification tests. EIS measurements were conducted using a 10 mM PBS solution containing 5.0 mM of the redox probe [Fe(CN)6]^3–/4–^ (Sigma-Aldrich UK, # 702587 and #60279) at a fixed potential of +0.125 V, using a sinusoidal perturbation with amplitude of 5 mV at a frequency range of 100,000–0.1 Hz.

**FIGURE 1:**
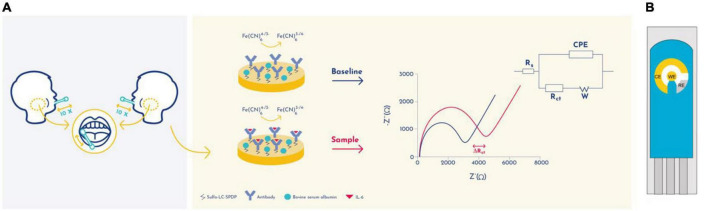
Schematic representation of the detection principle of the EIS-based immunosensor. **(A)** Randles circuit was used to fit the electrochemical impedance data: electrolyte resistance (Rs), constant phase element (CPE), Warburg element (W), and charge transfer resistance (Rct). **(B)** A schematic representation of a gold screen-printed electrode system. CE, Counter Electrode; RE, Reference electrode; WE, Working electrode.

Using the Nova software, the impedance data was fitted to a Randles equivalent circuit [Rs(CPE- [RctW])] (Randles, 1947; Cruz et al., 2019, 2021; Figure 1 insert). This circuit comprises the ohmic resistance of the electrolyte solution (R_*s*_) the Warburg impedance (W) resulting from the diffusion of the redox-probe, constant phase element (CPE), and the charge-transfer resistance (R_*ct*_) (Randles, 1947).

For the determination of the calibration curve and extrapolation of IL-6 concentrations, the extracted R_*ct*_ value was used. All EIS measurements were performed using a potentiostat/galvanostat, equipped with a Frequency Response Analysis module (Metrohm Autolab, PGSTAT302N/FRA32M) and controlled by NOVA software.

### Immunosensor analytical performance for IL-6 detection

Several IL-6 solutions (from 1 to 20 pg/mL), were prepared by diluting 200 pg/mL standard IL-6 solutions (eBioscience, #88-7066) in 0.9% NaCl, and used to calculate the IL-6 calibration curve. The antigen/antibody interaction occurs a during 30 min, followed by Milli-Q DI rinsing. The calibration curve was quantified based on the linear correlation between the normalized Rct value ((Rct(_*IL*–6_) - Rct(_*BSA average*_))/Rct(_*BSA average*_)) and the common logarithm (base 10) of IL-6 concentration. LOD corresponds to ((3SD_*blank*_ - b)/m), where SD_*blank*_ corresponds to the standard deviation of BSA Rct values. The m (slope) and b (the Y Intercept) values were obtained from the calibration curve. Different concentrations of recombinant TNFα cytokines (eBioscience, #88-7346) or in a cocktail solution with TNFα, IL-6 were used to test the specificity and selectivity of the immunosensor.

### Saliva sample collection and processing

Saliva samples were collected from 118 children, upon written informed consent obtained from the families.

Table 1 describes the characteristics of the sample. The sample had a similar proportion of males and females, and was predominantly Caucasian (98.4%), consistent with the general population, and the mean aged of assessment was 4.4 years old. Recruitment took place in preschools with children from families that varied in terms of psychosocial risk. The use of saliva samples was approved by the Portuguese Committee for Data Protection (Authorization number: 2496/2012) and the Ethical Committee of the University of Minho, Portugal (Authorization number: SECVS 027/2016). Informed consent was obtained from all participants included in this study according to ethical committee regulations. Salivette devices (Sarstedt, Rommelsdorft, Germany), following the manufacturer’s specifications were used to collect saliva samples. Children were instructed to place a cotton swab in the mouth and chew for a minute. Salivettes were kept chilled before being centrifuged at 4^°^C at 3,200 rpm for 10 min. Samples were aliquot and then stored at −80^°^C. An aliquot was used for each validation test.

**TABLE 1:** Descriptive information (*N* = 118).

	Mean ± SD or %	Range
**Children information**		
Children sex (% female)	50.8	
Mean age of assessment (years)	4.4 ± 0.7	3–6
Ethnicity (% Caucasian)	98.4	
CBCL internalization score (mean)	14.1 ± 8.0	1–47
CBCL externalization score (mean)	14.6 ± 6.8	3–40
CBCL total score (mean)	44.0 ± 20.2	9–131

### Enzyme-linked immunosorbent assay testing for IL-6 detection in human samples

IL-6 was measured, in human saliva samples (*n* = 119), using commercially available ELISA kits (eBioscience, #88-7066) according to the manufacturer’s specifications.

### Immunosensor for IL-6 quantification in saliva samples

Saliva samples were diluted 25% in 0.9% NaCl and the ΔR_*ct*_ determined upon the incubation of 1 μL of sample for 30 min in the WE, followed by rinsing with Milli-Q water. For the recovery test, 3 independent saliva samples were used, and tested in triplicates. For the immunosensor validation, saliva samples from 50 children (male or female) were used and tested in triplicates.

### Child emotional and behavioral problems

Emotional and Behavioral problems were assessed with the Child Behavior Checklist. (CBCL) (Achenbach and Rescorla, 2000; Achenbach et al., 2014). Caregivers completed the Portuguese version of the Child Behavior Checklist for children 1.5–5 years of age. For each of the 100 items that describe behavioral/emotional problems, the caregiver rated the child’s behavior on a 3-point scale: 0 = not true, 1 = sometimes/somewhat true, or 2 = very/frequently true. The CBCL shown to have good validity for the Portuguese version (Achenbach et al., 2014). When the score was equal or above 60 in the CBCL total scale, or 17 in the CBCL internalization or externalization score was considered positive for the presence of behavior problems. Table 1 describes the characteristics of the sample.

### Specificity and sensitivity of IL-6 biomarker

Children who are tested positive for behavior problems using CBCL (total, internalization or externalization) instrument and have detectable levels of IL-6 were defined true positives (TP); children’s without behavior problems using CBCL (total, internalization or externalization) who tested negative for presence of IL-6 were called true negatives (TN). Children with behavior problems who are tested negative for IL-6 are called false negatives (FN); Children’s without behavior problems who have tested positive for IL-6 are called false positives (FP). The sensitivity (true positive rate) was determine by:$\frac{T\,P}{\left(T\,P+F\,N\right)}$ and the specificity (true negative rate) was determine by: $\frac{T\,N}{\left(T\,N+F\,P\right)}$.

### Statistical analysis

GraphPad Prism Software version 6.0 (GraphPad Software Inc.) was used to performed statistical analysis. Pair *t*-test were used to compare differences between groups. Results are expressed as mean ± standard deviation. Differences were considered significant at *p* < 0.05.

## Results

### Impedimetric immunosensor for sensitive and quantitative IL-6 detection

In order to evaluate the presence of IL-6 in saliva samples, a gold screen-printed electrodes (SPE) has been developed with immuno-functionalized working electrode (WE) to transduce the biomarker concentration into electrochemical signals (Figure 1). The IL-6 quantification was performed by electrochemical impedance spectroscopy (EIS) analysis, which is a label and amplification free technique that allows direct molecular quantifications on modified surface electrodes (Sánchez et al., 2008). EIS is used for electrochemical-based biomarker detection as it is able of quantify alterations in electrical properties during working electrode (WE) surface modifications and biorecognition events (Figure 1B). Gold was selected as the material for the WE due to its good stability, biocompatibility and capacity to interact with cross-linking agents such as sulfo-LC-SPDP. Bovine serum albumin (BSA) was used to prevent nonspecific binding.

**FIGURE 2:**
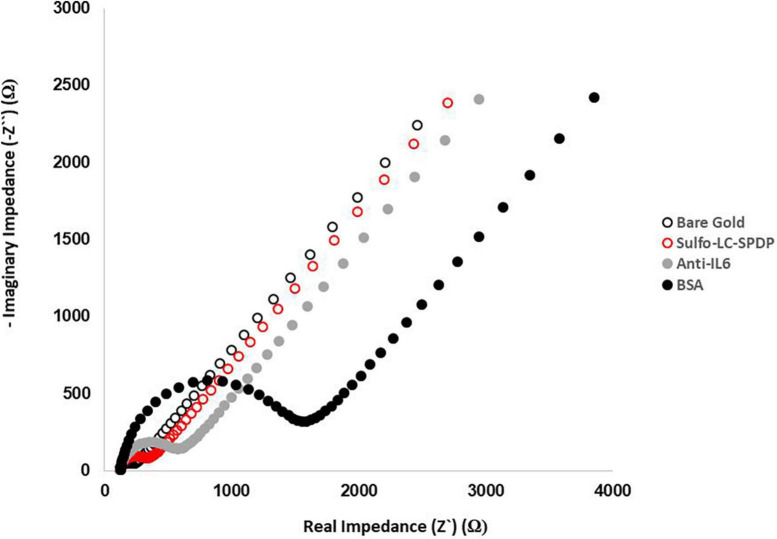
Evaluation of the functionalization steps by EIS. Nyquist plots of the functionalization process were obtained in 5.0 mM [Fe(CN)6]^3–/4–^ PBS buffer pH 7.4 using a sinusoidal potential perturbation of 5 mV over a frequency range of 1 × 10^5^−0.1 Hz.

The gold WEs were modified with a self-assembled monolayer (SAM) of sulfo-LC-SPDP to immobilize the antibody (Cruz et al., 2019, 2021) for the immunorecognition of IL-6 (Arya et al., 2013; Figure 1). Sulfo-LC-SPDP permits N-hydroxysuccinimide (NHS) ester group reaction with amines in the antibody forming a covalent bond, whereas the 2-mercaptopyridine acts as a spacer on the gold surface to facilitate electron transfer (Arya et al., 2013). The chemical modification of the gold WE was analyzed by EIS. Impedance are graphically represented as Nyquist plots and experimental data fitted to the modified Randles equivalent circuit (Figure 1). The charge-transfer resistance (R_*ct*_) is set by the semi-circle diameter obtained in EIS (Figure 1A). R_*ct*_ increase correlates with electron transfer blockage and therefore the successful modification of the WE surface, as seen in Figure 2, indicating the formation of the SAM and subsequent functionalization steps (anti-IL-6 and BSA immobilization). The average Rct and Rct variation between functionalization steps (ΔRct, i-1) for the total number of sensors used to achieve the calibration curve for each biomarker are presented in Table 2.

**TABLE 2:** Charge-transfer resistance values (Rct) and variations between the consecutive functionalization steps (Rct,i-1) with the respective standard deviation.

Modification step	Parameter	Biosensor (*n* = 6)
Bare gold	R_ct_ (Ω)	134.1 ± 24.4
	Δ R_ct_, i-1 (Ω)	–
Sulfo-LC-SPDP	R_ct_ (Ω)	226.2 ± 31.5
	Δ R_ct_, i-1 (Ω)	92.1 ± 7.1
Anti-IL-6 antibody	R_ct_ (Ω)	493.7 ± 70.6
	Δ R_ct_, i-1 (Ω)	267.5 ± 39.1
BSA	R_ct_ (Ω)	1291.0 ± 280.1
	Δ R_ct_, i-1 (Ω)	797.3 ± 209.5

### Immunosensor analytical performance

**FIGURE 3:**
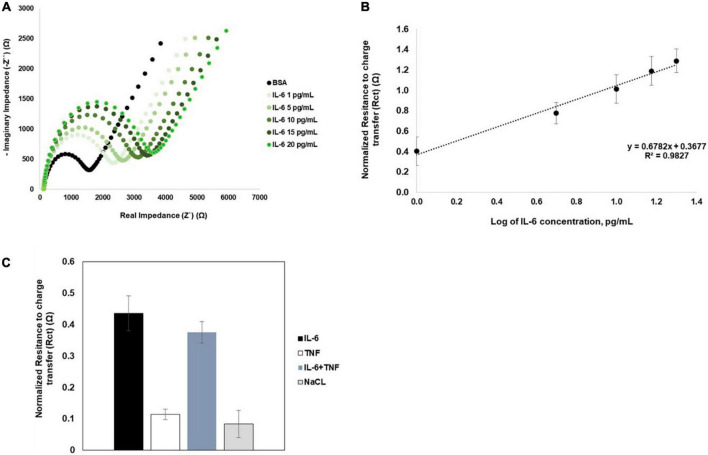
Nyquist plots and biosensor calibration curves. **(A)** Representative Nyquist plots of increasing concentrations of IL-6. **(B)** The calibration curves for IL-6 was obtained from an average of six independent sensors (*n* = 6). Error bars correspond to SD. **(C)** Immunosensor selectivity and specificity was analyzed by comparing normalized Rct values obtained for IL-6 or TNFa spiked in 0.9% NaCl; IL-6 spiked in a cocktail solution with TNFα, and for 0.9% NaCl solution (*n* = 5).

Previous studies indicate that saliva IL-6 concentrations in healthy individuals varies between of 0.7–10 pg/mL, whereas in different disease patients, the value is higher (Bougea et al., 2020; Corey-Bloom et al., 2020; Moreno-Quispe et al., 2020). Therefore, the immunosensor capacity to detect different concentrations of IL-6 was analyzed. Increasing IL-6 concentrations, were spiked in 0.9% NaCl solution and measured in individual sensors (*n* = 6). The immunosensor recognition response to increasing IL-6 concentrations is shown in the Nyquist plots (Figure 3A), with the increase in the semi-circle between concentration. The IL-6 immunosensor exhibited linear range between 1 and 20 pg/mL (Figure 3B). The linear regression is given by *y* = 0.6782*x*+0.3677 with *R*^2^ = 0.9827. The calculated limit of detection (LOD) was 0.088 pg/mL

The specificity of the immunosensor was assessed using IL-6 in 0.9% NaCl or in a 0.9% NaCl solution with TNFα (*n* = 5). The normalized R_*ct*_ values observed for IL-6 alone or in the cocktail solution with TNFα were not significantly different (Figure 3C). Additionally, the Rct variation for IL-6 was in the same variation range of R_*ct*_ measurements obtained for 0.9% NaCl alone. These results indicate that the immunosensor is highly selective and specific for IL-6.

Next the applicability of the immunosensor in detecting IL-6 in saliva samples collected from control healthy individuals (female and male) was tested. Initially the immunosensor was tested with non-pooled saliva samples (from 3 independent individuals, in triplicates each) spiked with different concentrations of IL-6. However, the immunosensor was unresponsive to increasing concentrations of recombinant IL-6, with R_*ct*_ values being unaffected with variations in the IL-6 concentrations. These results suggest that saliva matrix impacts immunosensor performance, and a sample preprocessing step is needed, as previously described for other body fluids (Cruz et al., 2019). This limitation is avoided by diluting the saliva sample in 0.9% NaCl. Saliva samples (from 3 independent individuals, in triplicates each) were diluted to 25% in 0.9% NaCl and spiked with different concentration of IL-6. As shown in Table 3, the biological matrix effect was minimized and the immunosensor is able to detect different concentration of IL-6 spiked in saliva samples, with recovery rate of at least 97.6%.

**TABLE 3:** IL-6 recovery tests in human saliva diluted at 25% in 0.9% NaCl (*n* = 3).

Spiked IL-6 (pg/mL)	Biosensor (mean ± SD)	Recovery rate (%)
2	2.0 ± 0.1	100.6 ± 4.8
5	5.0 ± 0.2	97.6 ± 7.0
10	10.2 ± 0.4	101.9 ± 3.6

### Immunosensor applicability to human saliva analysis

**FIGURE 4:**
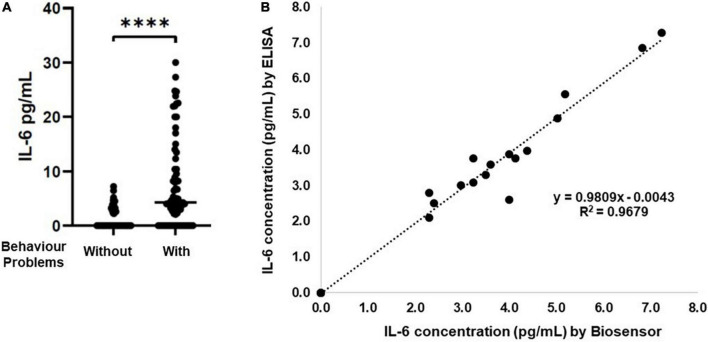
Correlation between Immunosensor and ELISA results for detection of IL-6 in saliva samples. **(A)** Saliva samples from children’s with different behavior problems were analyzed by conventional ELISA assay (*n* = 118). **(B)** Graphic representation of ELISA and immunosensor correlation. The dashed line shows the regression line with *R*^2^ = 0.9679, ****p > 0.0001.

The capacity of salivary inflammatory biomarkers to inform about systemic conditions is under evaluation (Rathnayake et al., 2013; Yoshizawa et al., 2013). Saliva samples are easy to collect in vulnerable patient populations like children’s (Rathnayake et al., 2013; Yoshizawa et al., 2013), being a non-invasive body fluid and alternative to blood. IL-6 an inflammatory cytokine is associated with the presence of mental health problems, such as depression or anxiety (O’Donovan et al., 2010; Trapero and Cauli, 2014; Raposa et al., 2015; de Baumont et al., 2019; Müller et al., 2019; Niraula et al., 2019), or internalization and externalization behaviors (Slopen et al., 2013; Tang et al., 2019). Additionally, higher levels of IL-6 in childhood are associated with increased risk of psychiatric disorders in adulthood (Khandaker et al., 2014; de Baumont et al., 2019; Perry et al., 2021). Consequently, highlighting the relevance of detecting IL-6 in human saliva samples from vulnerable populations such as children’s. Therefore, first IL-6 levels were quantified by ELISA in clinical saliva samples collected from children’s diagnose previously with behavior problems (using the CBCL instrument). Although in our cohort (composed of 50.8% female, with a range of 3–6 years), we do not observe differences in the IL-6 levels due to gender or age (Supplementary Figure 1), the results demonstrate that IL-6 levels are higher in saliva samples from children’s with behavior problems (Figure 4A).

Yet, a large number of samples were below detection limits of the ELISA assay (LOD = 2 pg/mL). The dynamic range of the ELISA assay is 2–200 pg/mL.

Accordingly, the optimized immunosensor was tested to detect IL-6 in saliva samples (*n* = 50). The accuracy of the immunosensor for the determination of IL-6 was analyzed by comparing the immunosensor and ELISA results to the same saliva sample. The linear correlation analysis was *R*^2^ = 0.9679 (Figure 4B), demonstrating that the results of each sample by both methods are in agreement with each other. Importantly, different IL-6 levels were possible to detect in saliva samples from children’s with behavior problems, that were not detected by the ELISA assay, due to the different LOD (Table 4), increasing the sensitivity of the detection of IL-6 in these samples from 28 to 90% (Table 5). Although, the specificity of the IL-6 biomarker for behavior problems identification is low (24%), the proposed immunosensor is able to increase the specificity of detection to 62% (Table 5). Additionally, the proposed immunosensor uses 50 times smaller sample volumes, and is 10 times faster than the ELISA assay. These results suggest that the label-free and real-time detection capabilities of the immunosensor can serve as a good immunoassay alternative to the laborious and time-consuming ELISA method.

**TABLE 4:** Example of different patterns obtained for IL-6 concentrations measured in saliva samples, accordingly to the different sensitivity of the ELISA and biosensor assays.

	ELISA	Biosensor
	
Sample	LOD: 2 pg/mL	LOD: 0.088 pg/mL
1	ND	ND
2	ND	ND
3	ND	ND
4	2.2 ± 0.1	2.1 ± 0.1
5	2.4 ± 0.1	2.5 ± 0.4
6	3.6 ± 0.5	3.6 ± 0.5
7	2.3 ± 0.7	2.1 ± 0.2
8	3.5 ± 0.4	3.3 ± 0.5
9	2.3 ± 0.2	2.8 ± 0.5
10	ND	2.2 ± 0.1
11	ND	1.5 ± 0.0
12	ND	1.3 ± 0.1
13	ND	2.0 ± 0.1
14	ND	1.4 ± 0.2
15	ND	1.6 ± 0.1

**TABLE 5:** IL-6 biomarker matrix used for specificity and selectivity analysis.

		Elisa	Biosensor
Behavior problems	TP	8	26
	FP	16	8
	FN	21	3
	TN	5	13
Specificity (%)		24	62
Sensitivity (%)		28	90

TP, true positive; FP, false positive; FN, false negative; TN, true negative.

## Discussion

Through a gold-based EIS immunosensor, a proof of concept protocol was developed to detect IL-6 in saliva samples. The immunosensor herewith validated is highly sensitive, with significantly lower LOD than conventional ELISA assays while maintaining the same specificity and consistency. Additionally, the proposed immunosensor holds the advantage of being easy to use, having low processing and signal acquisition times and requiring smaller samples volumes. Therefore, demonstrating a potentially effective point-of-care strategy for inflammatory biomarkers quantification in saliva, offering an attractive solution for the diagnosis of different systemic conditions, especially in vulnerable patient population such as children’s.

One of the huge throwback in the mental area, is the difficulty to have reliable accurate and objective methods to identify biomarkers that could help medical decisions (Hidalgo-Mazzei et al., 2018). This is especially important in mental illnesses since patients present enormous heterogeneity in their clinical presentation and are classified by diagnostic categories with a vast range of symptoms. Therefore, the identification of biomarkers, rather than using only behavioral symptoms and signs, might offer an additional precise tool for diagnosis, thereby supporting mental illnesses classification similarly to what is already used in other areas of medicine. In fact, it has been widely accepted that diagnosis based on one single biomarker may not provide sufficient accuracy (Xu et al., 2015). Therefore, the analysis of multiple biomarkers in association with clinical symptoms, may help clinicians to make a better diagnostic decision.

In line with this, the identification of good and reliable biomarkers for mental illness can only be responded with sampling plans that include several quantifications during the day (and across days), within the same individual and in “real life” contexts. The sampling requirement is particularly relevant in children’s, were collection of body fluids by non-invasive means is critical. Additionally, the near- and long-term health implications of identifying mental illness in children’s early in life is essential, particularly due to the public health costs of mental and physical disease, highlighting the need of more accurate diagnostic methodologies to help medical decisions. Therefore, a portable immunosensor, as the one described in this study, capable of detecting biomarkers in non-invasive fluids (e.g., saliva) rapidly, with high sensitivity and in decentralized settings with no requirements for specialized personnel, holds great potential for the identification and screening of multiple molecular biomarker pathways underlying the mechanistic complexity of mental illness.

Nevertheless, the study described here has some limitations, namely the lack of information and children’s cohort profiling concerning the different factors that could potentially influence IL-6 levels in children, such as the socio-economic adversities, the existence of health behaviors or body mass index. Despite the need for additional studies to assure the potential of IL-6 as a relevant biomarker for diagnosing and monitoring mental problems in children’s, in combination with other biomarkers and clinical symptoms, this work demonstrates that this immunosensor is pertinent to ensure this goal.

## Data availability statement

The raw data supporting the conclusions of this article will be made available by the authors, without undue reservation.

## Ethics statement

The studies involving human participants were reviewed and approved by the Portuguese Committee for Data Protection (Authorization number: 2496/2012) and the Ethical Committee of the University of Minho, Portugal (Authorization number: SECVS 027/2016). Informed consent was obtained from all participants included in this study according to ethical committee regulations. Written informed consent to participate in this study was provided by the participants’ legal guardian/next of kin.

## Author contributions

AC, IM-P and PF: conceptualization and formal analysis. AC and MV: methodology. AC: validation. AM, AS, and IS: clinical samples collection and selection. AC: writing—original draft preparation. AC, IS, IM-P, and PF: writing—review and editing. PF and IS: project administration. PF, IS and AM: funding acquisition. All authors have read and agreed to the published version of the manuscript.

## References

[B1] Achenbach T. M. Rescorla L. A. (2000). *Manual for the ASEBA preschool-age forms & profiles.* Burlington, VT: University of Vermont.

[B2] Achenbach T. Rescorla L. Dias P. Ramalho V. Lima V. S. Machado B. (2014). *Manual of the Achenbach system of empirically based assessment (ASEBA) to preschool and school ages.* Braga: Psiquilíbrios.

[B3] Arya S. K. Wang K. Y. Wong C. C. Rahman A. R. A. (2013). Anti-EpCAM modified LC-SPDP monolayer on gold microelectrode based electrochemical biosensor for MCF-7 cells detection. *Biosens. Bioelectron.* 41 446–451. 10.1016/j.bios.2012.09.006 23021854

[B4] Bariya M. Nyein H. Y. Y. Javey A. (2018). ‘Wearable sweat sensors’. *Nat. Electron.* 1 160–171. 10.1038/s41928-018-0043-y

[B5] Bougea A. Spantideas N. Galanis P. Katsika P. Boufidou F. Voskou P. (2020). ‘Salivary inflammatory markers in tension type headache and migraine: The SalHead cohort study’. *Neurol. Sci.* 41 877–884. 10.1007/s10072-019-04151-4 31823093

[B6] Brenhouse H. C. Schwarz J. M. (2016). ‘Immunoadolescence: Neuroimmune development and adolescent behavior’. *Neurosci. Biobehav. Rev.* 70 288–299. 10.1016/j.neubiorev.2016.05.035 27260127 PMC5412135

[B7] Calcia M. A. Bonsall D. R. Bloomfield P. S. Selvaraj S. Barichello T. Howes O. D. (2016). ‘Stress and neuroinflammation: A systematic review of the effects of stress on microglia and the implications for mental illness’. *Psychopharmacology* 233 1637–1650. 10.1007/s00213-016-4218-9 26847047 PMC4828495

[B8] Carpenter L. L. Gawuga C. E. Tyrka A. R. Lee J. K. Anderson G. M. Price L. H. (2010). Association between plasma IL-6 response to acute stress and early-life adversity in healthy adults. *Neuropsychopharmacology* 35 2617–2623. 10.1038/npp.2010.159 20881945 PMC2978751

[B9] Corey-Bloom J. Fischer R. S. Kim A. Snell C. Parkin G. M. Granger D. A. (2020). ‘Levels of Interleukin-6 in saliva, but not plasma, correlate with clinical metrics in Huntington’s disease patients and healthy control subjects. *Int. J. Mol. Sci.* 21:6363. 10.3390/ijms21176363 32887270 PMC7503233

[B10] Cruz A. Abreu C. M. Freitas P. P. Pinto I. (2021). *Impedimetric immunosensing for neuroinflammatory biomarker profiling, in neuromethods series.* Berlin: Springer Nature.

[B11] Cruz A. Queirós R. Abreu C. M. Barata C. Fernandes R. Silva R. (2019). ‘Electrochemical immunosensor for TNFα-mediated inflammatory disease screening’. *ACS Chem. Neurosci.* 10 2676–2682. 10.1021/acschemneuro.9b00036 30985099

[B12] Cuartas J. (2020). Heightened risk of child maltreatment amid the COVID-19 pandemic can exacerbate mental health problems for the next generation. *Psychol. Trauma* 12 S195–S196. 10.1037/tra0000597 32463282

[B13] Danese A. Moffitt T. E. Harrington H. Milne B. J. Polanczyk G. Pariante C. M. (2009). Adverse childhood experiences and adult risk factors for age-related disease. *Arch. Pediatr. Adolesc. Med.* 163 1135–1143. 10.1001/archpediatrics.2009.214 19996051 PMC3560401

[B14] de Baumont A. Bortoluzzi A. Aguiare B. W. Scotton E. Guimarães L. S. P. Kapczinski F. (2019). Anxiety disorders in childhood are associated with youth IL-6 levels: A mediation study including metabolic stress and childhood traumatic events. *J. Psychiatr. Res.* 115 43–50. 10.1016/j.jpsychires.2019.05.011 31103845

[B15] del Rey A. Besedovsky H. O. (2017). ‘Immune-neuro-endocrine reflexes, circuits, and networks: Physiologic and evolutionary implications. *Front. Horm. Res.* 48 1–18. 10.1159/000452902 28245448

[B16] D’Elia A. T. D. Matsuzaka C. T. Neto J. B. B. Mello M. F. Juruena M. F. Mello A. F. (2018). Childhood sexual abuse and indicators of immune activity: A systematic review. *Front. Psychiatry* 9:354. 10.3389/fpsyt.2018.00354 30127754 PMC6088139

[B17] Erta M. Quintana A. Hidalgo J. (2012). Interleukin-6, a major cytokine in the central nervous system. *Int. J. Biol. Sci.* 8 1254–1266. 10.7150/ijbs.4679 23136554 PMC3491449

[B18] Estévez P. T. Satoguina J. Nwakanma D. C. West S. Conway D. J. Drakeley C. J. (2011). ‘Human saliva as a source of anti-malarial antibodies to examine population exposure to *Plasmodium falciparum*’. *Malar. J.* 10:104. 10.1186/1475-2875-10-104 21527045 PMC3112448

[B19] Fagundes C. P. Way B. (2014). ‘Early-life stress and adult inflammation. *Curr. Dir. Psychol. Sci.* 23 277–283. 10.1177/0963721414535603

[B20] Gohel V. Jones J. A. Wehler C. J. (2018). ‘Salivary biomarkers and cardiovascular disease: A systematic review’. *Clin. Chem. Lab. Med.* 56 1432–1442. 10.1515/cclm-2017-1018 29630504

[B21] Gunnar M. Quevedo K. (2007). ‘The neurobiology of stress and development’. *Annu. Rev. Psychol.* 58 145–173. 10.1146/annurev.psych.58.110405.085605 16903808

[B22] Hänsel A. Hong S. Cámara R. J. A. von Kanel R. (2010). ‘Inflammation as a psychophysiological biomarker in chronic psychosocial stress’. *Neurosci. Biobehav. Rev.* 35 115–121. 10.1016/j.neubiorev.2009.12.012 20026349

[B23] Hidalgo-Mazzei D. Young A. H. Vieta E. Colom F. (2018). ‘Behavioural biomarkers and mobile mental health: A new paradigm’. *Int. J. Bipolar Disord.* 6:9. 10.1186/s40345-018-0119-7 29730832 PMC6161977

[B24] Jasim H. Carlsson A. Hedenberg-Magnusson B. Ghafouri B. Ernberg M. (2018). ‘Saliva as a medium to detect and measure biomarkers related to pain’. *Sci. Rep.* 8:3220. 10.1038/s41598-018-21131-4 29459715 PMC5818517

[B25] Jiang N. M. Cowan M. Moonah S. N. Petri W. A. Jr. (2018). ‘The Impact of systemic inflammation on neurodevelopment’. *Trends Mol. Med.* 24 794–804. 10.1016/j.molmed.2018.06.008 30006148 PMC6110951

[B26] Jiao W. Y. Wang L. N. Liu J. Fang S. F. Jiao F. Y. Pettoello-Mantovani M. (2020). Behavioral and emotional disorders in children during the COVID-19 epidemic. *J. Pediatr.* 221 264.e–266.e. 10.1016/j.jpeds.2020.03.013 32248989 PMC7127630

[B27] Khandaker G. M. Pearson R. M. Zammit S. Lewis G. Jones P. B. (2014). ‘Association of serum interleukin 6 and C-reactive protein in childhood with depression and psychosis in young adult life’. *JAMA Psychiatry* 71:1121. 10.1001/jamapsychiatry.2014.1332 25133871 PMC4561502

[B28] Lacey R. E. Kumari M. Bartley M. (2014). ‘Social isolation in childhood and adult inflammation: Evidence from the national child development study’. *Psychoneuroendocrinology* 50 85–94. 10.1016/j.psyneuen.2014.08.007 25197797

[B29] Loades M. E. Chatburn E. Higson-Sweeney N. Reynolds S. Shafran R. Brigden A. (2020). Rapid systematic review: The impact of social isolation and loneliness on the mental health of children and adolescents in the context of COVID-19. *J. Am. Acad. Child Adolesc. Psychiatry* 59 1218.e–1239.e. 10.1016/j.jaac.2020.05.009 32504808 PMC7267797

[B30] Mesquita A. R. Belsky J. Crego A. Fachada I. Oliveira P. Sampaio A. (2015). ‘Neural correlates of face familiarity in institutionally reared children with distinctive, atypical social behavior. *Child. Dev.* 86 1262–1271. 10.1111/cdev.12371 25899924

[B31] Miller G. E. Chen E. Parker K. J. (2012). ‘Psychological stress in childhood and susceptibility to the chronic diseases of aging: Moving towards a model of behavioral and biological mechanisms’. *Psychol. Bull.* 137 959–997. 10.1037/a0024768.Psychological PMC3202072 21787044

[B32] Moreno-Quispe L. A. Serrano J. Virto L. Sanz M. Ramírez L. Fernández-Castro M. (2020). Association of salivary inflammatory biomarkers with primary Sjögren’s syndrome. *J. Oral Pathol. Med.* 49 940–947. 10.1111/jop.13070 32538490

[B33] Müller N. Krause D. Barth R. Myint A. Weidinger E. Stettinger W. (2019). ‘Childhood adversity and current stress are related to pro- and anti-inflammatory cytokines in major depression’. *J. Affect. Disord.* 253 270–276. 10.1016/j.jad.2019.04.088 31063941

[B34] Napodano C. Callà C. Fiorita A. Marino M. Taddei E. Cesare T. D. (2021). ‘Salivary biomarkers in COVID-19 patients: Towards a wide-scale test for monitoring disease activity’. *J. Pers. Med.* 11:385. 10.3390/jpm11050385 34066701 PMC8151878

[B35] Niraula A. Witcher K. G. Sheridan J. F. Godbout J. P. (2019). ‘Interleukin-6 induced by social stress promotes a unique transcriptional signature in the monocytes that facilitate anxiety’. *Biol. Psychiatry* 85 679–689. 10.1016/j.biopsych.2018.09.030 30447911 PMC6440848

[B36] O’Donovan A. Hughes B. M. Slavich G. M. Lynch L. Cronin M. O’Farrelly C. (2010). ‘Clinical anxiety, cortisol and interleukin-6: Evidence for specificity in emotion–biology relationships’. *Brain Behav. Immun.* 24 1074–1077. 10.1016/j.bbi.2010.03.003 20227485 PMC4361085

[B37] Perry B. I. Zammit S. Jones P. B. Khandaker G. M. (2021). ‘Childhood inflammatory markers and risks for psychosis and depression at age 24: Examination of temporality and specificity of association in a population-based prospective birth cohort’. *Schizophr. Res.* 230 69–76. 10.1016/j.schres.2021.02.008 33684738 PMC8224182

[B38] Prasad S. Tyagi A. K. Aggarwal B. B. (2016). ‘Detection of inflammatory biomarkers in saliva and urine: Potential in diagnosis, prevention, and treatment for chronic diseases’. *Exp. Biol. Med.* 241 783–799. 10.1177/1535370216638770 27013544 PMC4950402

[B39] Randles J. E. B. (1947). ‘Kinetics of rapid electrode reactions’. *Discuss. Faraday Soc.* 1 11–19. 10.1039/DF9470100011

[B40] Rapado-González Ó Martínez-Reglero C. Salgado-Barreira A. Takkouche B. López-López R. Suárez-Cunqueiro M. M. (2020). Salivary biomarkers for cancer diagnosis: A meta-analysis. *Ann. Med.* 52 131–144. 10.1080/07853890.2020.1730431 32056455 PMC7877992

[B41] Raposa E. B. Bower J. E. Hammen C. L. Najman J. M. Brennan P. A. (2015). ‘Inflammation: The role of negative health behaviors’. *Psychol. Sci.* 25 1268–1274. 10.1177/0956797614530570.A PMC4207738 24760142

[B42] Rathnayake N. Åkerman S. Klinge B. Lundegren N. Jansson H. Tryselius Y. (2013). ‘Salivary biomarkers for detection of systemic diseases’. *PLoS One* 8:e61356. 10.1371/journal.pone.0061356 23637817 PMC3634781

[B43] Sánchez S. Sánchez S. Roldán M. Pérez S. Fàbregas E. (2008). ‘Toward a Fast, easy, and versatile immobilization of biomolecules into carbon nanotube/polysulfone-based biosensors for the detection of HCG hormone. *Anal. Chem.* 80 6508–6514. 10.1021/ac7025282 18662016

[B44] Simons R. L. Woodring D. Simons L. G. Sutton T. E. Lei M. Beach S. R. H. (2019). Youth adversities amplify the association between adult stressors and chronic inflammation in a domain specific manner: Nuancing the early life sensitivity model. *J. Youth Adolesc.* 48 1–16. 10.1007/s10964-018-0977-4 30603835 PMC7685217

[B45] Slopen N. Kubzansky L. D. Koenen K. C. (2013). ‘Internalizing and externalizing behaviors predict elevated inflammatory markers in childhood’. *Psychoneuroendocrinology.* 38 2854–2862. 10.1016/j.psyneuen.2013.07.012 24011503

[B46] Tang A. Fox N. A. Nelson C. A. Zeanah C. H. Slopen N. (2019). ‘Externalizing trajectories predict elevated inflammation among adolescents exposed to early institutional rearing: A randomized clinical trial’. *Psychoneuroendocrinology.* 109:104408. 10.1016/j.psyneuen.2019.104408 31442936 PMC6842705

[B47] Ting E. Y.-C. Yang A. C. Tsai S.-J. (2020). ‘Role of interleukin-6 in depressive disorder’. *Int. J. Mol. Sci.* 21:2194. 10.3390/ijms21062194 32235786 PMC7139933

[B48] Trapero I. Cauli O. (2014). ‘Interleukin 6 and cognitive dysfunction’. *Metab. Brain Dis.* 29 593–608. 10.1007/s11011-014-9551-2 24782046

[B49] Wei W.-J. Mu S. Heiner M. Fu X. Cao L. J. Gong X. F. (2012). YB-1 binds to CAUC motifs and stimulates exon inclusion by enhancing the recruitment of U2AF to weak polypyrimidine tracts. *Nucleic Acids Res.* 40 8622–8636. 10.1093/nar/gks579 22730292 PMC3458536

[B50] Xu T. Fang Y. Rong A. Wang J. (2015). Flexible combination of multiple diagnostic biomarkers to improve diagnostic accuracy data analysis, statistics and modelling. *BMC Med. Res. Methodol.* 15:94. 10.1186/s12874-015-0085-z 26521228 PMC4628350

[B51] Yoshizawa J. M. Schafer C. A. Schafer J. J. Farrell J. J. Paster B. J. Wong D. T. W. (2013). ‘Salivary biomarkers: Toward future clinical and diagnostic utilities. *Clin. Microbiol. Rev.* 26 781–791. 10.1128/CMR.00021-13 24092855 PMC3811231

