# Supplementary figures and images for: Non-invasive diagnosis and monitoring tool of children’s mental health: A point-of-care immunosensor for IL-6 quantification in saliva samples
Source: Front Neurosci. 2022 Sep 26;16:919551. doi: 10.3389/fnins.2022.919551 (PMC9549322; doi:10.3389/fnins.2022.919551)

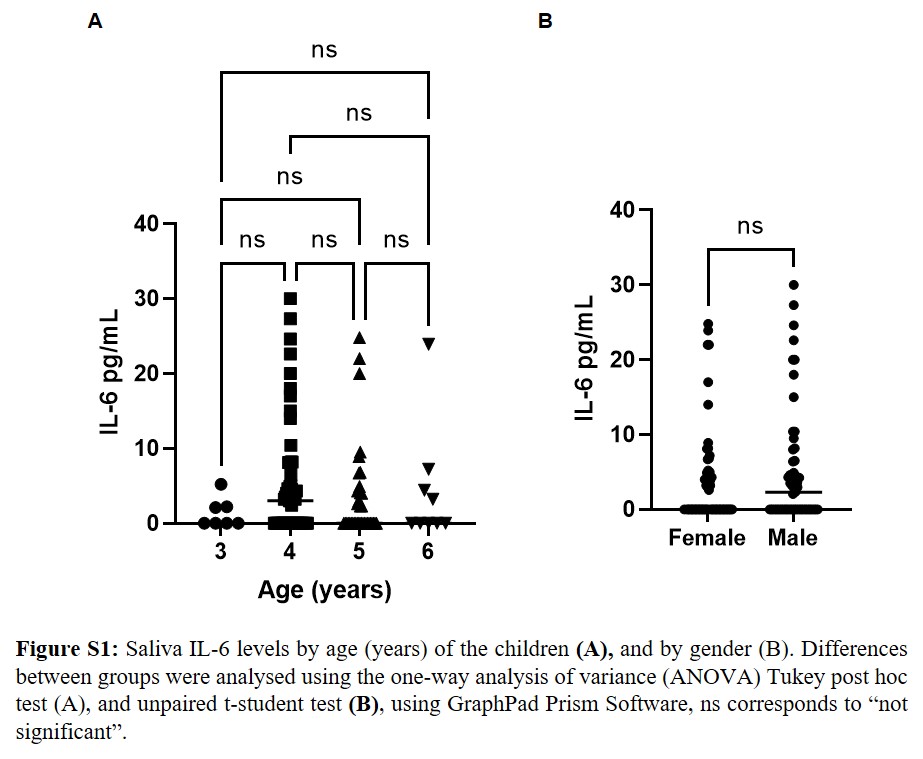

Supplement: Supplementary file 1 [file Image_1.jpg]
